# RNAseq Analysis of the Response of *Arabidopsis thaliana* to Fractional Gravity Under Blue-Light Stimulation During Spaceflight

**DOI:** 10.3389/fpls.2019.01529

**Published:** 2019-11-26

**Authors:** Raúl Herranz, Joshua P. Vandenbrink, Alicia Villacampa, Aránzazu Manzano, William L. Poehlman, Frank Alex Feltus, John Z. Kiss, Francisco Javier Medina

**Affiliations:** ^1^Plant Microgravity Lab, Centro de Investigaciones Biológicas (CSIC), Madrid, Spain; ^2^Department of Biology, University of North Carolina at Greensboro, Greensboro, NC, United States; ^3^School of Biological Sciences, Louisiana Tech University, Ruston, LA, United States; ^4^Department of Genetics and Biochemistry, Clemson University, Clemson, SC, United States

**Keywords:** Arabidopsis, fractional gravity, microgravity, stress response, RNA-Seq, spaceflight

## Abstract

**Introduction:** Traveling to nearby extraterrestrial objects having a reduced gravity level (partial gravity) compared to Earth’s gravity is becoming a realistic objective for space agencies. The use of plants as part of life support systems will require a better understanding of the interactions among plant growth responses including tropisms, under partial gravity conditions.

**Materials and Methods:** Here, we present results from our latest space experiments on the ISS, in which seeds of *Arabidopsis thaliana* were germinated, and seedlings grew for six days under different gravity levels, namely micro-*g*, several intermediate partial-*g* levels, and 1*g*, and were subjected to irradiation with blue light for the last 48 h. RNA was extracted from 20 samples for subsequent RNAseq analysis. Transcriptomic analysis was performed using the HISAT2-Stringtie-DESeq pipeline. Differentially expressed genes were further characterized for global responses using the GEDI tool, gene networks and for Gene Ontology (GO) enrichment.

**Results:** Differential gene expression analysis revealed only one differentially expressed gene (AT4G21560, VPS28-1 a vacuolar protein) across all gravity conditions using FDR correction (q < 0.05). However, the same 14 genes appeared differentially expressed when comparing either micro-*g*, low-*g* level (< 0.1*g*) or the Moon *g*-level with 1*g* control conditions. Apart from these 14-shared genes, the number of differentially expressed genes was similar in microgravity and the Moon *g*-level and increased in the intermediate *g*-level (< 0.1*g*), but it was then progressively reduced as the difference with the Earth gravity became smaller. The GO groups were differentially affected at each *g*-level: light and photosynthesis GO under microgravity, genes belonged to general stress, chemical and hormone responses under low-*g*, and a response related to cell wall and membrane structure and function under the Moon *g*-level.

**Discussion:** Transcriptional analyses of plants under blue light stimulation suggests that root blue-light phototropism may be enough to reduce the gravitational stress response caused by the lack of gravitropism in microgravity. Competition among tropisms induces an intense perturbation at the micro-*g* level, which shows an extensive stress response that is progressively attenuated. Our results show a major effect on cell wall/membrane remodeling (detected at the interval from the Moon to Mars gravity), which can be potentially related to graviresistance mechanisms.

## Introduction

Long-term exploration of the Solar System will require that humans travel within a nearly close life-support systems, reducing to the minimum the amount of water, oxygen, and nutrients to be transported and optimizing the recycling of reusable waste. Such a system is being under development, for example, in the Melissa project from the European Space Agency (Godia et al., 2004), but it will require an edible plant to be successfully cultivated in the environmental conditions expected to be achieved during spaceflight and on arrival at nearby objects. Isolation chambers could avoid, at least partially, some of the suboptimal environmental conditions that can greatly compromise organism adaptation to spaceflight (Beckingham, 2010), including temperature, radiation, air, and soil composition constrains. However, providing artificial gravity will require large diameter centrifuge (Van Loon et al., 2008) or even expensive railroad-based platforms that could be subjected to other biological limitations.

Gravity influences the direction of plant growth and the pattern of development, from seedlings to adult plants (Volkmann and Baluska, 2006), and even gravitational effects on *in vitro* plant cell cultures have been reported (Babbick et al., 2007; Barjaktarovic et al., 2007). Light is the only tropistic response that plays a substantial role in determining overall plant architecture with a contribution similar to gravity. Typically, plants orient their roots towards the gravity vector (positive gravitropic response), and away from blue/white light exposure (negative phototropic response). Shoots show the opposite orientation, growing away from the gravity vector (negative gravitropic response) and towards a blue/white light source (positive phototropic response; Kutschera and Briggs, 2016; Briggs, 2014; Chen et al., 1999). Any tropistic response is divided into three stages; perception, transduction and response. During the perception phase, starch-filled statoliths interact with other cellular components in the specialized columella cells. Once the gravity signal is perceived, a differential auxin gradient develops along the root to the root elongation zone (transduction stage), where differential plant growth occurs and leads to reorientation of the root in the direction of the gravity vector (reviewed in Vandenbrink et al., 2014).

Phototropism and gravitropism have been well characterized, but little was known about the interaction among tropisms until recently. Experiments with plants in microgravity have allowed for the study of phototropism in the absence of the influence of gravity (Kiss, 2015). Our previous research showed that light perception by the roots can have an effect on shoot gravitropism in *Arabidopsis thaliana* (Hopkins and Kiss, 2012). In addition, phototropic curvature of roots in response to unilateral blue light was tied to the magnitude of the gravity vector (Vandenbrink et al., 2016). This latter study also identified an association between red-light-based phototropism in roots and the magnitude of the gravity vector. Other experiments involving assays of cell growth and cell proliferation have demonstrated that there is an imbalance between these key plant development functions in microgravity (Matía et al., 2010) in dark-grown plants. Recent spaceflight results also demonstrated that red light can compensate this effect (Valbuena et al., 2018), particularly increasing cell growth (i.e., as assayed by ribosome biosynthesis in the nucleolus) that was depleted without light stimulation.

Studies on the response of living organisms to altered gravity are greatly facilitated by the development of ground-based facilities for simulation of gravity alterations to perform basic science as well as to design and prepare for space experiments (Herranz et al., 2013). The biological system (cell proliferation and growth during early plant development) had previously been studied under real microgravity in the ISS (Driss-Ecole et al., 2008; Matía et al., 2010; Mazars et al., 2014). Similar effects to the ones observed during spaceflight (increased cell proliferation rates together with decreased cell growth parameters) also were observed in root meristem cells in simulated microgravity studies (Bouchern-Dubuisson et al., 2016; Valbuena et al., 2018) and in two partial-*g* paradigms. The imbalance of the cell proliferation and cell growth rates is also observable at the Moon´s gravity level, while less pronounced effects were observed at Mars *g* level (Kamal et al., 2018; Manzano et al., 2018).

In terms of spaceflight transcriptional experiments, a number of studies performed in orbit are available in public databases (Paul et al., 2012; Paul et al., 2013; Johnson et al., 2017; Zupanska et al., 2017). In these studies, some Gene Ontology categories as responses to biotic or abiotic stress, oxidative stress, cell wall reorganization and secondary metabolism remodeling commonly show the highest variation in gene expression. Transcriptional response in microgravity is different in each experiment, due to both the biological material (developmental stage or organ analyzed) and the technical/environmental constraints of each spaceflight experiment [late or early access to the sample during spaceflight, the hardware used, type of dissection/fixation/preservation see for example (Kruse et al., 2017)].

In a previous report, we focused on the effects of microgravity in the transcriptional profile of blue-light photostimulated seedlings (Vandenbrink et al., 2019). Here, we will describe the plant transcriptional response to several partial gravity levels in young blue-light photostimulated *A. thaliana* seedlings cultivated into the European Module Cultivation System (EMCS) centrifuge on board the International Space Station (ISS). The results from plants cultivated on the ISS within the SEEDLING GROWTH experiment series illustrate the adaptation strategy of plants at the level of the whole transcriptome to cope with reduced gravity conditions.

## Materials and Methods

### Seedling Growth Spaceflight Experiments

Seeds of *A. thaliana* ecotype *Landsberg erecta* (Ler) were flown to the ISS *via* the SpaceX Dragon. Spaceflight experiments were conducted utilizing the European Modular Cultivation System (EMCS) in the Columbus Module of the ISS. The EMCS facility provides two centrifuges for creation of simulated gravity vectors, as well as atmospheric, temperature and hydration monitoring and control (Brinckmann and Schiller, 2002; Brinckmann, 2005; Kiss et al., 2014). In addition, the EMCS contains a video camera for image acquisition as well as monitoring of growth. The Seedling Growth series of experiments was conducted in two parts. The first set of seedlings were uploaded on SpaceX CRS-2 (March 2013) followed by return *via* CRS-3 (May 2014), and the second set of seedlings were carried to the ISS on SpaceX CRS-4 (September 2014) and returned on CRS-5 (February 2015).

### Spaceflight Procedures

Experimental containers were uploaded to the ISS and loaded into the EMCS as previously described (Kiss et al., 2014; Vandenbrink and Kiss, 2016; Vandenbrink et al., 2019). Experimental conditions were controlled remotely from the Norwegian User Support and Operations Centre (N-USOC; Trondheim, Norway). The experiment was initiated *via* hydration of the seeds. Plants were grown under 6 nominal gravity conditions produced by different rotational speeds on the EMCS centrifuge, microgravity (stopped EMCS centrifuge), 0.1, 0.3, 0.5, 0.8, and 1.0 *g*. The angular speed to generate each fractional gravity level was calculated for the cassette in the center of the Experimental Container. In the case of the 1.0 g cassettes, the value was calculated for the fifth cassette in order to prevent values higher than Earth nominal gravity. Seedlings were illuminated under white light (30–40 µmol m^−2^ s^−1^) for 96 h, followed by 48 h of unidirectional photostimulation with blue light. Light sources were LEDs (Kiss et al., 2014). RNA-Seq analysis was only conducted on seedlings exposed to unidirectional blue light. After conclusion of the experiments, seedlings were frozen in dedicated holders by placing them at -80°C in the General Laboratory Active Cryogenic ISS Experiment Refrigerator (GLACIER) freezer of the ISS. Upon return of frozen seedlings to Earth, samples were transported on dry ice and immediately preserved with RNALater for subsequent RNA-Seq analysis.

### RNA Extraction and Sequencing

RNA was extracted individually for each EC TROPI cassette for most of the samples (i.e. from 24 cassettes, 20 samples were obtained to collect approximately 10–15 seedlings per extraction). A plant specific RNA extraction NucleoSpin kit (MACHEREY-NAGEL, Catalog # 740949.250) including a DNase treatment was used to isolate whole plant mRNA. The quantity and quality of the extracted RNA was determined by Nanodrop 2000 (Thermo Scientific). Extracted RNA was keep frozen at −80 C until shipped on dry ice to the David H. Murdoch Research Institute in Kannapolis; North Carolina, USA. During sequencing, twenty total RNA samples were used to generate twenty sequencing libraries using the Illumina TruSeq RNA Library Preparation Kit (Illumina, USA). Samples were individually indexed. The samples then were combined at equimolar proportions into three pools with 6–7 samples per pool. Each pool was loaded onto a single lane of a flow cell. A 125bp paired end sequencing run was performed on the Illumina HiSeq2500.

Paired-end 125bp reads were aligned to the *Arabidopsis* TAIR10 genomes using the HISAT2 pipeline on the Clemson University Palmetto Cluster (Kim et al., 2015). Fragments with a Phred score below 33 were filtered using Trimmomatic (Trimmomatic, 2013). HISAT2 (*v2.1.0*) was used to align sequencing reads. Reads were assembled into transcripts using StringTie (*v1.3.4*). Annotation was conducted using TAIR10 FASTA sequence the TAIR10 genome GTF annotation file (www.arabidopsis.org). This transcriptional dataset has been submitted to the GENELAB database (https://genelab.nasa.gov) and it will be released with the reference GLDS-251.

### Differential Gene Expression Analysis

Statistical analyses of differential gene expression was conducted utilizing DESeq2 (v1.18.1; Anders and Huber, 2010). A multiple-test corrected p-value (q-value; Benjamini and Hochberg, 1995) of 0.05 was employed. The 20 samples were organized to reduce the *g*-level interval within biological replicates so the following groups were established: microgravity (stopped EMCS centrifuge, 4 replicates), low gravity (0.09 ± 0.02*g*, 3 replicates), Moon gravity (0.18 ± 0.04*g*, 3 replicates), Mars gravity (0.36 ± 0.02*g*, 3 replicates), reduced Earth gravity (0.57 ± 0.05*g*, 4 replicates) and 1*g* control (0.99 ± 0.06*g*, 3 replicates). Venn Diagrams comparing the number of differentially expressed genes (DEG) across gravity levels were created using jvenn [http://jvenn.toulouse.inra.fr/app/index.html (Bardou et al., 2014)] with both q-value < 0.05 and p-value < 0.05. Afterwards, gene ontology (GO) analysis of specific groups of DEGs was performed using BinGO (Maere et al., 2005) with the full list of GO terms (GO_Full) or using PANTHER (Mi et al., 2019) with the molecular functions, biological process and cellular component GO lists. Subcellular localization of DEGs was analyzed using the abundance tool (MMAP) of the Subcellular Localization Database for Arabidopsis Proteins [SUBA4, (Hooper et al., 2017)].

For a global view of the whole genome transcriptional status along *g*-levels into the SG1 and SG2 experiment (comparisons versus 1*g*), global expression patterns were calculated using the Gene Expression Dynamics Inspector (GEDI v2.1) program analysis (Eichler et al., 2003). GEDI profile allows the visualization of the gene expression across the transcriptome generating a mosaic image or dot matrix, consisting of 5 x 9 pixels (average of 5–14 probe sets/tile) using a self-organizing map algorithm and standard setting of the software (Eichler et al., 2003). Analysis was done using the signal log2 ratio of the selected probe sets through using the 5,571 probes with any significant (p < 0.05) change in expression from more than 21,000 sequences assignated to annotated genes. The same study was repeated adding the false discovery rate correction (FDR, q < 0.05, 861 genes). Each pixel represents a group or cluster of genes that share a similar transcriptional profile in any experimental condition. Each pixel has a color which reflects the average expression of the genes included in the cluster for each experimental condition compared to 1*g* control in each panel. The GEDI program SOM algorithm determines which genes should be assigned to each cluster, and then places similar clusters in a nearby area of the mosaic, creating an image and allowing global transcriptome analysis as a single entity for display in different gravitational conditions. For certain pixels of interest, the gene list extracted from the clusters was used to find functional links between genes using Genemania App embedded into the Cytoscape v3.6.1 (Shannon et al., 2003) software with default settings.

## Results

### Identification of Differentially Expressed Genes (DEG)

**Figure 1:**
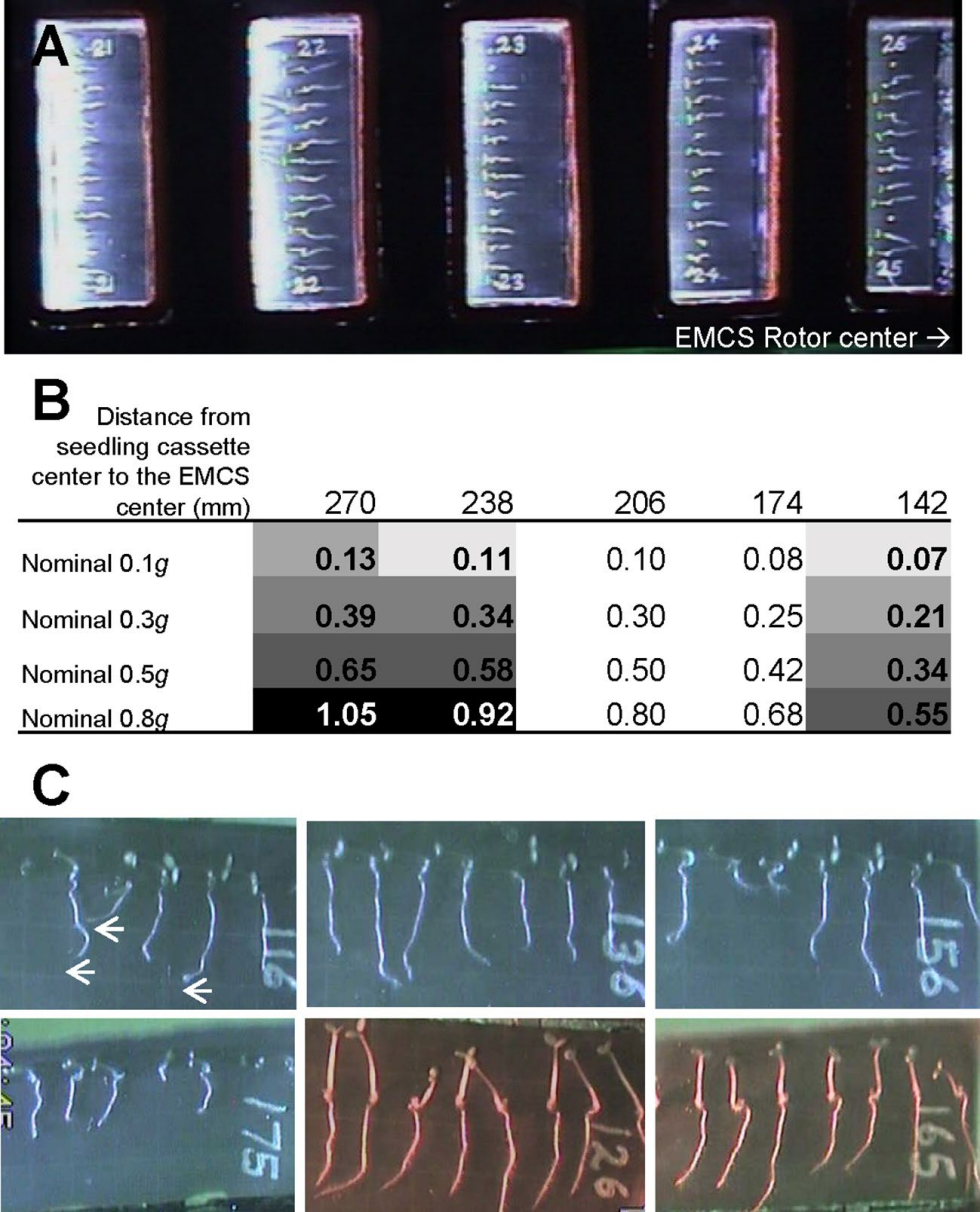
Setup of SG1/SG2 experiment on board the International Space Station. **(A)** Image of an experimental container with 5 seedling cassettes inside the European Modular Cultivation System (EMCS) with the direction to the EMCS rotor center included. **(B)** Calculated *g*-level in each of the five culture chambers depending on the distance to the EMCS centrifuge rotor (note that only samples in the 3 positions in bold were included in this analysis, cassettes at 174 and 206 mm from EMCS center did not contain wildtype samples) and different EMCS rotational speed (nominal *g* value). Different background grey tones are used to indicate the samples that were used as replicates for low gravity (0.09 ± 0.02*g*, 3 replicates), Moon gravity (0.18 ± 0.04*g*, 3 replicates), Mars gravity (0.36 ± 0.02*g*, 3 replicates) and reduced Earth gravity (0.57 ± 0.05*g*, 4 replicates) and 1*g* control (0.99 ± 0.06*g*, 3 replicates) in addition to the microgravity samples (stopped centrifuge, 4 replicates). **(C)** Closer view of 6 day-old seedlings growing within a seed cassette at microgravity (CC116), low *g* (0.07*g*, CC136), Moon *g* (0.21*g*, CC156) or 1*g* control (1.05*g*, CC175) conditions with blue light stimulation (from the left). Hypocotyls show a clear positive phototropism at any *g*-level but roots only show this tropism at microgravity (arrows). For comparison, seed cassettes at low *g* (0.07*g*, CC126) and 1*g* control (1.05*g*, CC165) conditions exhibiting root positive phototropism to red light stimulation (from the left) are provided (see Vandenbrink et al., 2016 for a detailed phototropism discussion).

We performed transcriptomic studies with young seedlings of *A. thaliana* that were grown on the ISS at different gravity levels depending on the rotational speed of the EMCS centrifuge (nominal *g* levels) and the distance of each cassette to the rotation center (**Figure 1**). Seeds were hydrated to initiate our spaceflight experiment as previously described (Kiss, 2015; Vandenbrink and Kiss, 2019) showing a positive blue-light phototropism in the microgravity samples that it is greatly reduced at 0.1*g*, and effectively negated at 0.3*g* and higher gravity levels (Vandenbrink et al., 2016). Root growth was also determined after blue-light stimulation. The results show longer roots in microgravity and 1*g* samples in comparison with 0.1*g* seedlings (Vandenbrink et al., 2016).

**Figure 2:**
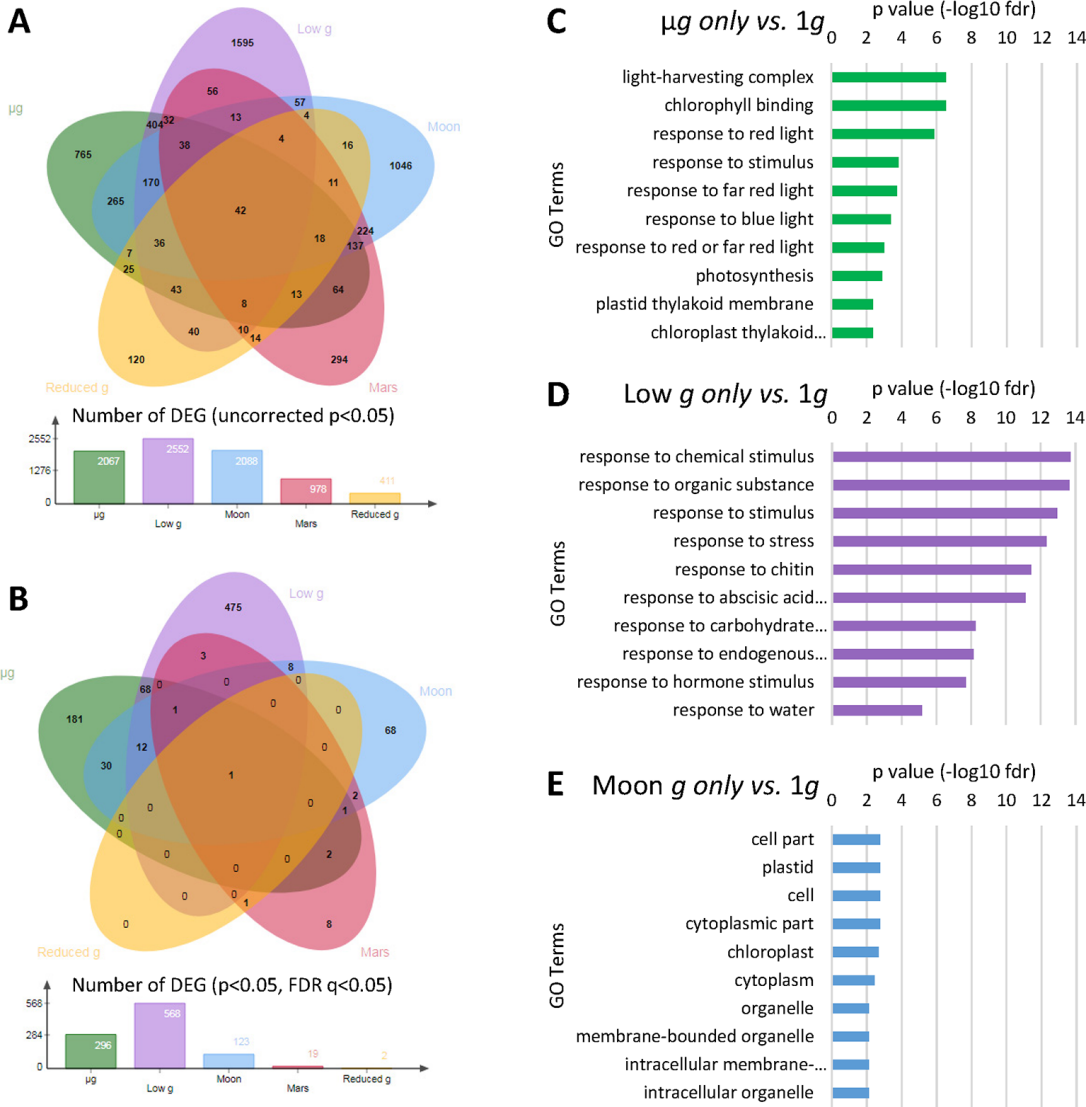
Differentially expressed genes (DEGs) across the different gravity levels. **(A)** Venn diagram classifying DEGs using uncorrected p-value (p < 0.05). **(B)** Venn diagram classifying DEGs including an adjusted FDR q-value (p and q < 0.05). **(C)** Ten most significant gene ontology (BinGO GO Full Enrichment) categories from DEG in µ*g* vs. 1*g* only (FDR q < 0.05). **(D)** Ten most significant gene ontology (BinGO GO Full Enrichment) categories from DEG in low gravity (circa 0.1*g*) vs. 1*g* only (FDR q < 0.05). **(E)** Ten most significant gene ontology (BinGO GO Full Enrichment) categories from DEG in Moon gravity vs. 1*g* only (FDR q < 0.05).

Differential expression analysis was conducted *via* DESeq2 (Anders and Huber, 2010) among all five reduced gravity conditions taking into account the calculated *g*-level experienced in each EC due to the geometry of the EMCS container (**Figure 1B**), using Earth’s gravity (1*g*) as the reference group, extending previous results from the microgravity samples (Vandenbrink et al., 2019). Initially, a reduced stringency analysis was done to isolate all genes identified as differentially expressed with a p-value of p < 0.05. Comparison between µ*g* and 1*g* revealed 2067 differentially expressed genes, comparisons between low gravity (lower than 0.1*g)* and 1*g* peaked at 2552 genes, comparisons between Moon *g-level* and 1*g* reduced to 2088 genes, comparisons between Mars *g-level* and 1*g* revealed 978 genes, and lastly, comparisons between reduced Earth *g*-level (0.57*g*) and 1*g* identified only 411 differentially expressed genes (**Figure 2A**). In addition to an uncorrected p-value of p < 0.05, a stringent Benjamini and Hochberg (1995) FDR q-value <0.05 was used in the identification of differentially expressed genes (**Figure 2B**). Comparison between µ*g* and 1*g* revealed 296 differentially expressed genes, while fractional gravity comparisons between low gravity (lower than 0.1*g*) and 1*g* revealed 568 genes. Comparisons between Moon *g*-level and 1*g* revealed 123 genes and comparisons between Mars *g*-level and 1*g* revealed 19 genes. Lastly, comparisons between reduced Earth *g*-level (0.57*g*) and 1*g* identified only 2 differentially expressed genes. Only one DEG appeared in all reduced gravity conditions (AT4G21560, VPS28-1 a vacuolar protein sorting homolog gene), another in all but reduced Earth *g* level (AT5G45428). There were 12 common DEG in microgravity, low *g* and Moon *g* conditions, being most of them related with calcium signaling, redox status and stress response (**Supplementary Table 1**).

The ten most significant GO terms (BinGO full GO terms list) of the genes differentially expressed (obtained with the adjusted q-value in **Figure 2B**) specifically expressed in µ*g*, low-*g*, or Moon gravity only were identified (**Figures 2C–E**). Not only the number of significantly affected genes, but also the type of genes affected, were clearly different with the increasing partial *g* level. In microgravity, we observed an enrichment in GO terms related with light and photosynthesis. In low gravity, there was a quite global stress effect together with chemical and hormone responses. Finally, when plants are grown under the Moon gravity level, the more representative enrichment is related to cell wall and membrane structure and function related genes. In fact, the differential subcellular localization of the DEGs at the Moon *g* level shows a clear enrichment in plastid related genes and other cell wall/membrane systems, while the general stress response observed at the low *g* level is characterized by the very large unassigned Subcellular compartment group (**Supplementary Figure 1** and **Supplementary Table 2**).

### Partial Gravity Differential Effect

**Figure 3:**
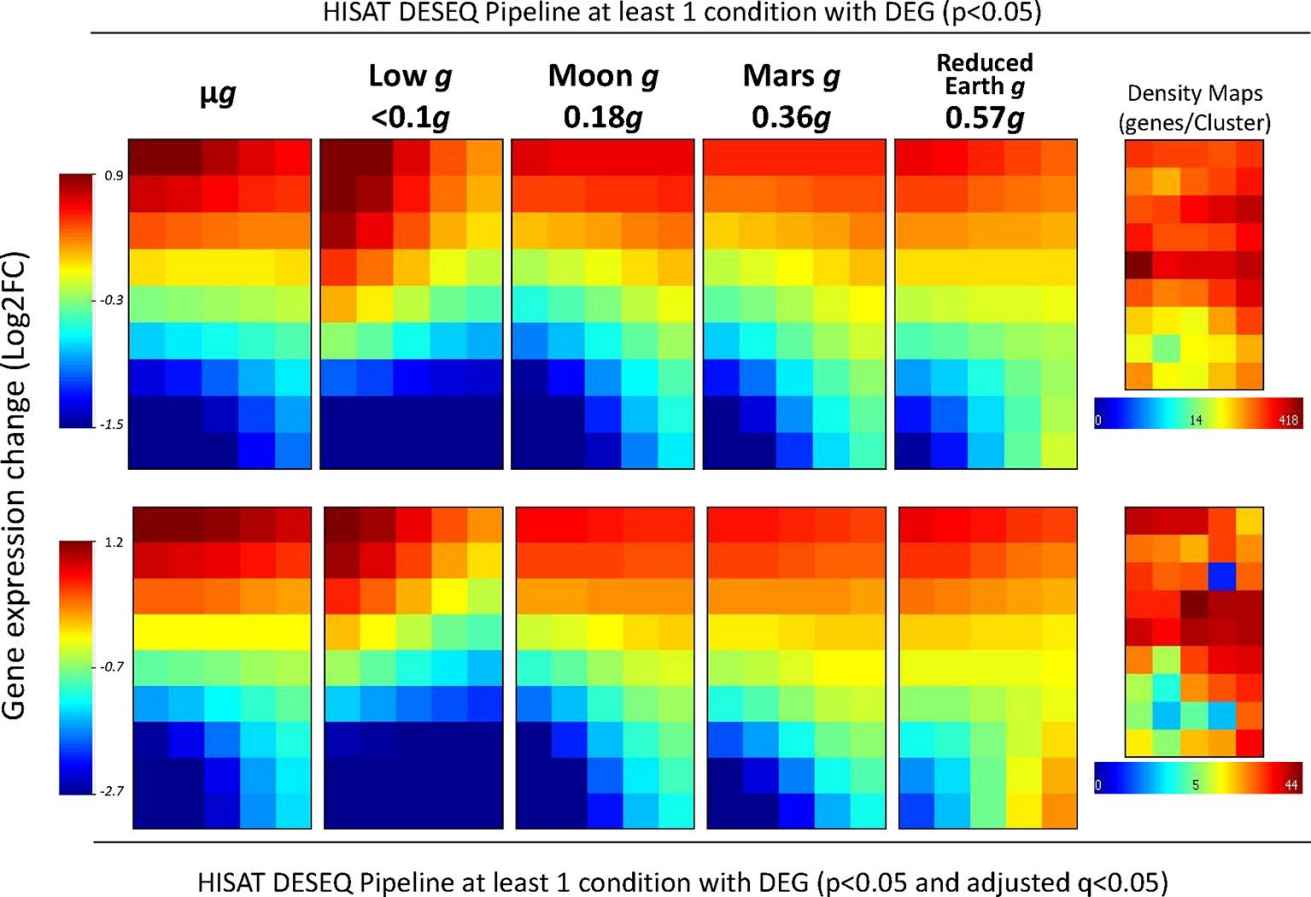
Whole-genome transcriptional status variations along *g*-levels into the SG1/SG2 experiments (comparisons versus 1*g*). A 5x9 clustering analysis of the differentially expressed genes (in at least one of the partial *g* levels, using normal *p* values (first row, n = 5,571 genes) or adjusted *q*-values (second row, n = 861 genes) on the transcriptome have been done with the average of each gene expression level within a similarly expressed cluster across the samples. Values are shown according to the log2 ratio scales at the border of the figure (from highly overrepresented in red to highly down-represented in blue). The mean value of −0.3 or −0.7 indicates an overall repression in gene expression under microgravity. The gene density maps are shown in the middle of the figure for each analysis. Calculated *g*-levels have been obtained by considering replicates the more similar samples across the nominal µ*g*, 0.1*g*, 0.3*g*, 0.5*g*, 0.8*g* and 1*g* (precisely, µ*g* (4 replicates), 0.09 ± 0.02*g* (3 replicates), Moon level (0.18 ± 0.04*g*, 3 replicates), Mars-level (0.36 ± 0.02*g*, 3 replicates), 0.57 ± 0.05*g* (4 replicates) and control 1*g* (0.99 ± 0.06*g*, 3 replicates).

Although the effects of partial-*g* on gene expression (at the levels of the Moon or Mars) appeared limited in the first analysis (**Figure 2**), we then evaluated how the expression recovers to normal values from microgravity to 1*g* condition by using a visual tool that creates a mosaic image for each *g*-level representing the gene expression level of similarly behaving DEG (in at least one of the conditions, n = 5571, p < 0.05 without FDR correction, **Figure 3** first row). Except in the case of the low *g* condition, it is clear that the areas in red (up-regulated gene clusters) and the areas in blue (down-regulated genes clusters) that appear in the microgravity panels became quantitative and qualitatively smaller with increasing *g*-level. In the case of the low *g* condition, different clusters and with greater expression changes appears, suggesting an overlapping of two different responses at <0.1*g* level. The same result is shown if we apply the FDR correction (n = 861, p < 0.05 and q < 0.05 FDR correction, **Figure 3** second row).

**Figure 4:**
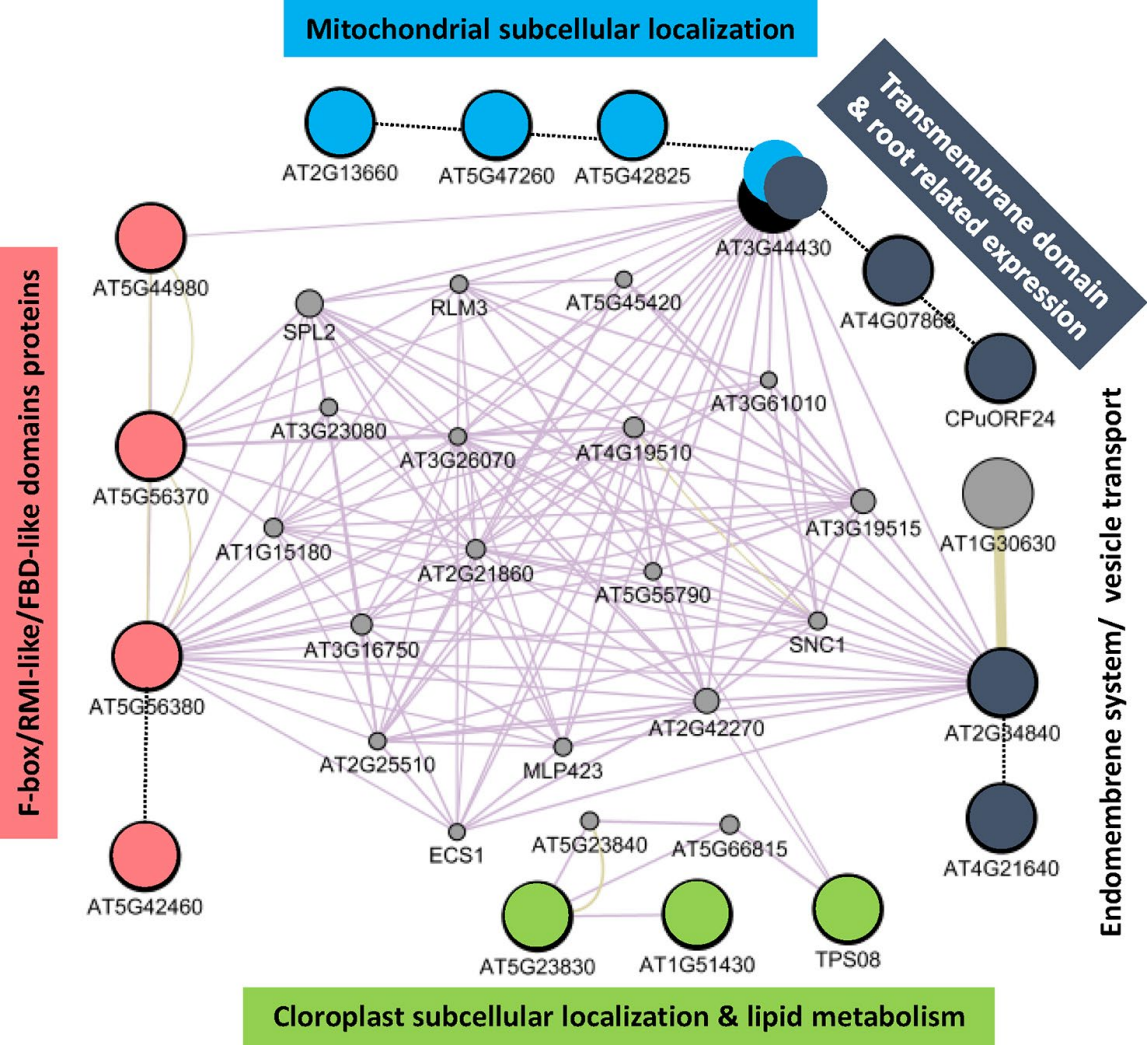
Fifteen up-regulated genes in reduced gravity conditions can be connected in a gravity-response putative pathway. Large circles shown the genes detected in the first row clusters from the previous GEDI analysis (**Figure 3**) and small circles are candidates to be members of a shared pathway (as detected by Genemania App in Cytoscape v3.6.1). While most of the genes have unknown function, color have been used to highlight shared features among their GO properties. Solid lines shown related features as detected by Genemania app, dotted lines have been added by manual datamining comparisons. Note that four genes expressing F-box/RMI-like proteins (in red) out of 156 genes (related examples are auxin polar transport genes as TIR-1, cell wall remodeling and even cyclins) in the genome may be key in the definition of a microgravity specific pathway characterized by the highly interconnected nodes in this graph.

Additionally, we took advantage of the GEDI self-organizing maps to select the list of commonly upregulated genes due to reduced gravity from the clusters in the first row of the GEDI panels. When the list of obtained genes (**Supplementary Table 3**) is used as a query in GeneMANIA, a tool to create networks from gene database content, a putative pathway for gravity response is proposed. Several processes related to mitochondria, plastid, cell wall and cell membrane processes are clearly affected together with 4 proteins (out of 156 annotated in the genome) belonging to the F-box/RMI-like/FDB-like domain family (including members as *TIR-1* auxin signaling gene, cell wall remodeling and even cyclins, **Figure 4**).

**Figure 5:**
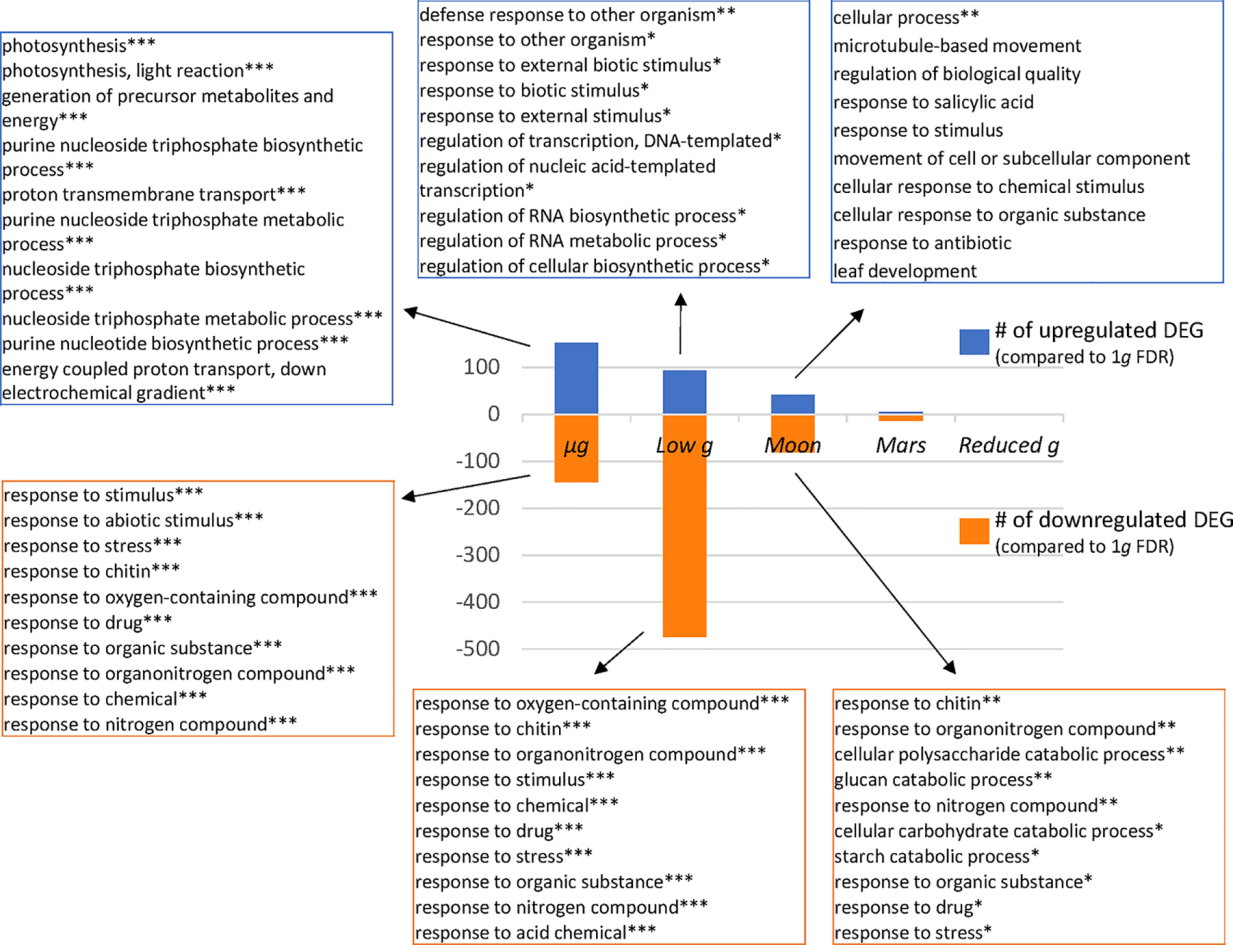
Biological processes affected by reduced gravity (Panther Enrichment in biological process GO). Total number of up-regulated and down-regulated DEG (p < 0.05) are shown for the five reduced gravity conditions vs. 1*g* control. The ten most significant gene ontology (Panther biological process GO Enrichment) categories for the three upregulated and the three downregulated DEGs are shown for the microgravity, low gravity (circa 0.1*g*) and Moon gravity (0.18*g*) vs. 1*g* (*** FDR q < 0.001, **FDR q < 0.01, *FDR q > 0.05).

Finally, in an attempt to further dissect the differential response to partial gravity, we analyzed separately the up-regulated and down-regulated DEGs (**Figure 5** and **Supplementary Table 2**). We can observe a general pattern in which the number of DEG fades with increasing *g* level but with a remarkable exception. In the list of down-regulated DEGs without FDR correction there is an unusually high number of affected genes in both low *g* and Moon *g* level (not shown). These DEGs are more clearly confirmed only in low *g* after FDR correction, in which more than 50% of the non-corrected DEG remains significant after the FDR correction. These genes belong to stress related GO terms, particularly related with the accessibility to plant nutrients (**Figure 5**).

## Discussion

### Blue Light Phototropism May Be Enough to Reduce the Gravitational Stress Response on Orbit

Image analysis of seedlings grown during the Seedling Growth suite of experiments previously characterized a novel blue-light phototropic response in roots of *A. thaliana* grown in conditions of microgravity (Vandenbrink et al., 2016). This relationship was shown to be linearly related to the magnitude of the gravity vector for plants exposed to red light, but plants exposed to blue light showed rapid attenuation of the response in the presence of increasing gravity levels. To determine the differential response to reduced gravity and attempt to dissect the molecular mechanisms of gravisensing, we performed RNA-seq analysis to characterize changes in gene expression that may be associated with the novel blue phototropic response. Interestingly, the effect of blue-light illumination is clearly observable in the microgravity samples, with a clear enrichment in GO terms related with light perception, photosynthesis and biosynthesis of the photosynthetic complexes as previously reported (Vandenbrink et al., 2019), but it is barely appearing in the GO enrichment analyses that we performed on partial gravity samples, even bellow 0.1*g* (low *g* conditions). Surprisingly, the phototropic response to the blue light seems to be enough to cancel the effects of other genes of interest in gravitational research (as the ones observed at low *g* or Moon *g* level) to be not significantly affected in microgravity conditions. This result may be complementing previous reports from the same Seedling Growth spaceflight experiment but with plants exposed to red photostimulation. Fundamental plant functions, as cell proliferation and cell growth activity in root meristems, known to be affected by microgravity in the absence of light (Matía et al., 2010) are balanced just by providing a red photostimulation phase (Valbuena et al., 2018). In both red and blue photostimulation samples within the Seedling Growth experiment, roots are exhibiting a positive phototropism that can compensate the gravitropism stimuli role that it is required to preserve meristematic competence in orbit, as shown by the longer root growth in microgravity and 1*g* samples in comparison with 0.1*g* seedlings (Vandenbrink et al., 2016).

### Low-*g* Effect: Competition Between Tropisms or Artifacts of Reduced-Gravity Simulation?

The large number of DEGs detected in the low-*g* conditions (<0.1*g*) is a striking result of this study. The type of transcriptional response observed in this group is similar to stress-related responses reported in other spaceflight or simulated microgravity datasets (Paul et al., 2012; Correll et al., 2013; Sugimoto et al., 2014; Ferl et al., 2015; Kwon et al., 2015; Johnson et al., 2017; Paul et al., 2017; Shi et al., 2017; Zupanska et al., 2017; Choi et al., 2019). In this case, there is a very clear component of “Response to General Stress” with a FDR q value <10^−10^, without any other GO terms to be similarly affected. In contrast the responses at the microgravity level (FDR q value <10^−6^), or at the Moon *g*-level (FDR q value <10^−2^) are very subtle. We explain these results as being the consequence of the combination of two tropistic responses acting with very low intensity. It is very likely that the subtle blue phototropism and the weak gravitropism signal at approximately 0.09*g* are competing to take the leading role in providing the fundamental cue for driving seedling growth and plant development. The result is a stress for the plant (blue-LEDS are located laterally while the gravity vector is towards the bottom of the cassette), which needs to adapt its developmental plan to an environment without the usual tropistic cues. The transcriptional adaptation to provide a response to this evolutionary novel and challenging environment requires the modification of more than five hundred genes, while microgravity only requires half of this number.

An alternative explanation to the low-*g* effects that cannot be completely excluded could be put in connection with simulated microgravity experiments. Secondary effects of microgravity simulation facilities (shear or inertial forces) and even small variations in the environmental conditions of experimental and control samples may lead to gene expression variations in a similar set of genes as those observed in the low-*g* subgroup in this work. Similarly, it is important to take into account the existence of hardware effects when growing plants in real microgravity (Kiss, 2015), including lack of convection, reduced CO_2_ levels, improper temperature, elevated ethylene, spacecraft vibrations, increased radiation exposure, among others.

However, the tropism conflict interpretation introduced early on this section seems to have a greater contribution than the artifacts of centrifugation in the low-*g* effects. A hardware side-effect explanation is less conceivable since the present study was conducted utilizing the European Modular Cultivation System, which contains an air scrubbing/filtration system designed for removing excess ethylene from the seedlings during the growth phase (Kiss et al., 2014; Kiss, 2015). Thus, even with proper ventilation, a reduction in gene expression of photosynthetic genes was observed in the microgravity samples (Vandenbrink et al., 2019). In the case of the samples exposed to centrifugation, the ventilation effect should be less important, but the centrifugation by itself could lead to additional stress in the samples. However, this centrifugation factor is also present in the 1*g* control sample, exposed to even higher angular speeds.

Spaceflight experimentation is required to verify that simulation strategies on Earth analogues are reliable and worthy. The continuous validation of the best simulation strategies will optimize and increase our chances of success in future spaceflight experiments (Herranz et al., 2013). Additional research in simulated low-*g* conditions on Earth or even in the Moon surface will help to extend and validate this research work.

### Moon and Mars-*g* Effects and the Consequences for Manned Spaceflight Missions

Cultivating plants as part of life support systems in nearby objects of our planet will require us to expose the plants to the partial-*g* interval within the two values we examined here, namely, Moon *g*-level (0.18 ± 0.04*g*) and Mars gravity (0.36 ± 0.02*g*). Although some of the genes and GO terms observed affected at lower *g*-levels also appeared in these conditions, the existence of the gravitropic response, in combination with blue light illumination, seems to be enough to restore a nearly normal transcriptional state, particularly at the Mars *g*-level. These results are consistent with previous data coming from partial gravity simulation paradigms that validated that *Arabidopsis* developmental plan is still affected at the Moon *g*-level (even more intensely affected than in similar simulated microgravity samples) but that the “normal” developmental plan is almost completely restored at Mars *g*-levels (Kamal et al., 2018; Manzano et al., 2018).

The identity of some of the GO terms significantly affected at the Moon *g*-level suggest some structural stress at the level of the cell wall and membrane systems. This result is consistent with other results in spaceflight experiments (Kwon et al., 2015; Johnson et al., 2017; Zupanska et al., 2017). This effect is progressively weaker from Moon-*g* (almost similar to microgravity) to Mars-*g*, which shows a less intense response, but it is still visible at *g*-levels as high as 0.57*g*, when utilizing the less stringent analysis (**Figure 2A**). At these *g*-levels, gravitropism response may be acting and suppressing the recently described blue-light root phototropism, (already at the Moon *g*-level, see **Figure 1C**).

Therefore, we suggest that this transcriptional response could be related with the graviresistance signal that the cells without professional gravisensing organelles (as the statolith in the columella cells of the root) may use to detect *g*-force (Soga, 2013). Particularly at the Moon *g*-level, the very weak gravitropic signal may be still in conflict with a graviresistance tension in the cell wall and membrane systems (also weak), inducing and additional stress that is progressively removed when Mars *g*-level is reached. In that regard, we have found that some of the genes changing throughout the series of reduced gravity levels can be connected in a pathway in which certain genes may have a central position (**Figure 4**). The most connected nodes in the pathway would be the genes involved in the cross-talk between the cell-membrane-localized (AT3G44430 and AT2G34840) and the F-box/RMI-like/FDB-like domain proteins (AT5G56370 and AT5G56380) candidate genes. An important caveat to our results is that RNA was obtained from whole seedlings, despite our assumption that the root is the organ that can discriminate better the weak phototropism and gravitropism signals that are proposed here to be responsible of the transcriptional variations we have shown here.

## Conclusions

The results of this study take advantage of the induction of subtle blue-light phototropism in roots in spaceflight to discern the transcriptional responses to different tropisms in orbit. Removal of the influence of gravity on blue-light-illuminated seedlings showed a reduction in gene expression in multiple pathways associated with photosynthesis, suggesting shared molecular pathways between the two tropistic responses, or a functional compensation among them.

It is important to emphasize that the effects shown at microgravity here are gradually removed by increasing *g*-load. While the phototropic effect is noted at the microgravity level, a general stress response is detected at <0.1*g*, probably due to conflicting stimuli, just at the detection threshold of photo- and gravi-sensing mechanisms. Membrane-related gene ontologies became the more significant at the Moon *g*-level, and they become progressively weaker at higher *g*-levels, allowing us to discriminate the differential contribution of the classical statolith-based gravitropism from other responses based on cell tensegrity that may require a higher *g*-threshold to be fully active (Hoson et al., 2005; Hoson and Wakabayashi, 2015). Therefore, our results are starting to isolate, at the whole transcriptional level, the global effects that are produced by the gravitropism, phototropism and graviresistance mechanisms, working at different *g*-level thresholds. Future use of mutant lines will help us to confirm and extend these findings, which suggests an intricate connection between gravity and light perception in *A. thaliana*. In the long term, these results on the interaction among tropisms will be important for the use of plants in bioregenerative support systems needed for the human exploration of the Solar System.

## Data Availability Statement

The datasets generated for this study can be found in the GeneLab database https://genelab-data.ndc.nasa.gov/genelab/accession/GLDS-251/.

## Author Contributions

RH and JV designed this partial gravity experiment within the Seedling Growth and wrote the manuscript. JV, WP and FF performed the RNA seq including a preliminary processing of the data. RH, AV and AM performed GEDI and GO analysis. JK and FM designed and coordinated the whole Seedling Growth project series of experiments on board ISS and contributed writing the manuscript. All authors reviewed and agree with the manuscript content.

## Funding

This work was supported by the Spanish “Plan Estatal de Investigación Científica y Técnica e Innovación” of the Ministry of Economy, Industry and Competitiveness (Grant numbers AYA2012–33982 and ESP2015–64323-R to FJM, cofounded by ERDF), by pre-doctoral fellowships to (AM) and (AV) from the Spanish National Program for Young Researchers Training (MINECO, Ref. BES-2013-063933, BES-2016-077976) and the Seedling Growth Project to the ISS LSRA2009-0932/1177 of ESA-ELIPS Program. Funding for the RNA-seq was provided by NASA Grants NNX12A065G and 80NSSC17K0546 to JZK. We acknowledge support of the publication fee by the CSIC Open Access Publication Support Initiative through its Unit of Information Resources for Research (URICI).

## Conflict of Interest

The authors declare that the research was conducted in the absence of any commercial or financial relationships that could be construed as a potential conflict of interest.
