# Supplementary material for: RNAseq Analysis of the Response of Arabidopsis thaliana to Fractional Gravity Under Blue-Light Stimulation During Spaceflight
Source: Front Plant Sci. 2019 Nov 26;10:1529. doi: 10.3389/fpls.2019.01529 (PMC6889863; doi:10.3389/fpls.2019.01529)
Supplement: Supplementary file 1 [file DataSheet_1.pdf]

Supplementary Table 1. List of the fourteen DEG (adjusted FDR q<0.05) in at least the three lower gravity conditions (microgravity, low *g* and Moon *g* level, extracted from Figure 2A analyses) as generated by GeneMANIA tool (Cytoscape).

Name	Description
KIC (AT2G46600)	Calcium-binding EF-hand family protein [Source:TAIR;Acc:AT2G46600]
AT5G54490	pinoid-binding protein 1 [Source:TAIR;Acc:AT5G54490]
RHL41 (AT5G59820)	C2H2-type zinc finger family protein [Source:TAIR;Acc:AT5G59820]
ATMKK9 (AT1G73500)	MAP kinase kinase 9 [Source:TAIR;Acc:AT1G73500]
CML38 (AT1G76650)	calmodulin-like 38 [Source:TAIR;Acc:AT1G76650]
AT3G50800	unknown protein, molecular function and cellular component; INVOLVED IN: N-terminal protein myristoylation; BEST Arabidopsis thaliana protein match is: unknown protein (TAIR:AT5G66580.1); [Source:TAIR;Acc:AT3G50800]
AT3G46620	zinc finger (C3HC4-type RING finger) family protein [Source:TAIR;Acc:AT3G46620]
ATERF-1 (AT4G17500)	ethylene responsive element binding factor 1 [Source:TAIR;Acc:AT4G17500]
AT-HSFA8 (AT1G67970)	heat shock transcription factor A8 [Source:TAIR;Acc:AT1G67970]
AT4G02540	Cysteine/Histidine-rich C1 domain family protein [Source:TAIR;Acc:AT4G02540]
ALPHA-DOX1 (AT3G01420)	Peroxidase superfamily protein [Source:TAIR;Acc:AT3G01420]
ERF104 (AT5G61600)	ethylene response factor 104 [Source:TAIR;Acc:AT5G61600]
CPuORF24 (AT5G45428)	conserved peptide upstream open reading frame 24 [Source:TAIR;Acc:AT5G45428]
VPS28-1 (AT4G21560)	vacuolar protein sorting-associated protein 28 homolog 1 [Source:TAIR;Acc:AT4G21560]

Supplementary Figure 1. Subcellular compartments are differently affected in reduced gravity conditions. Number of DEGs (uncorrected p<0.05) are represented for the five reduced gravity levels versus 1*g* control highlighting the subcellular compartment associated with those genes (see color code). Note the proportion of unassigned genes (higher than 50%) particularly in the Low *g* condition and the plastid-related genes at the Moon *g* level.

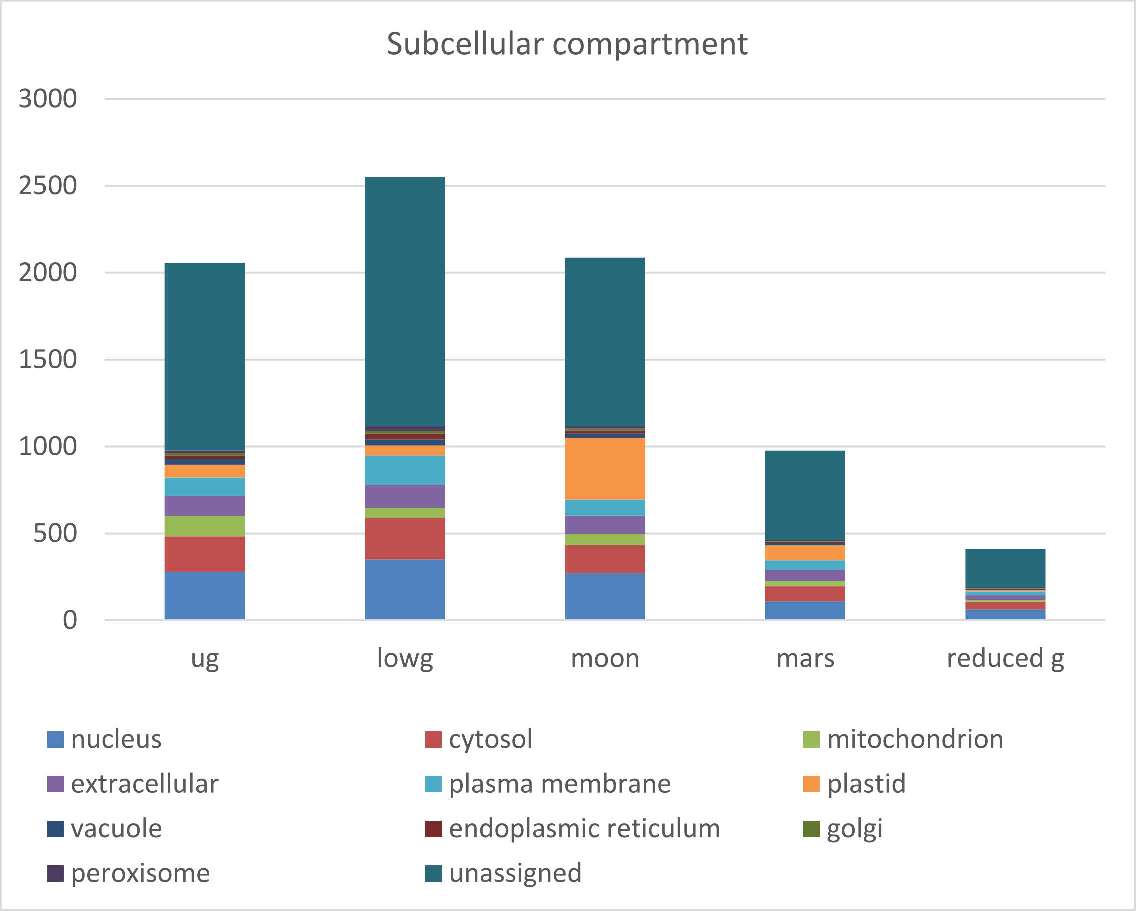

Supplementary Table 2. PANTHER-based GO overrepresentation test in the DEGs (FDR q<0.05) reduced gravity samples vs 1*g* control. Only GO terms with an enrichment raw p-value <0.01 were listed. A) Cellular component GO subanalysis (a lower raw p<0.05 was accepted at the Moon *g* level upregulated list, in *italics*). B) Biological Process GO subanalysis (additional FDR enrichment p<0.05 was applied in all conditions except the Moon *g* level upregulated list, in *italics*). C) Molecular Function GO subanalysis. D) Genelist used for the GO subanalysis (upregulated and downregulated DEGs (FDR q<0.05) under micro*g* /Low*g* / Moon *g* reduced gravity samples vs 1*g* control).

(ATTACHED AS EXCEL FILE VERSION)

Supplementary Table 3. List of the fifteen DEG (adjusted FDR q < 0.05) from upregulated GEDI clusters in all low gravity conditions (extracted from figure 3 analyses) as generated by GeneMANIA tool (Cytoscape). See a graphical representation in figure 4.

Name	Description
AT4G21640	Subtilase family protein [Source:TAIR;Acc:AT4G21640]<br>Related with Peptidase, proteinase inhibitor function
AT4G07868	unknown protein, molecular function and biological process; LOCATED IN: endomembrane system; [Source:TAIR;Acc:AT4G07868] TM protein domains.<br>Related with expression in siliqua and elongation zone root
AT5G56380	F-box/RNI-like/FBD-like domains-containing protein [Source:TAIR;Acc:AT5G56380]
AT2G34840	Coatomer epsilon subunit [Source:TAIR;Acc:AT2G34840]<br>Related with vesicules trasnsport between Golgi-ER
AT5G56370	F-box/RNI-like/FBD-like domains-containing protein [Source:TAIR;Acc:AT5G56370]
AT3G44430	unknown protein, molecular function and biological process; LOCATED IN: mitochondrion; EXPRESSED IN: 20 plant structures; EXPRESSED DURING: 13 growth stages; BEST Arabidopsis thaliana protein match is (TAIR:AT5G41660.1); [Source:TAIR;Acc:AT3G44430] TM protein domains
AT5G42825	unknown protein [Source:TAIR;Acc:AT5G42825] Related with mitochondrion
AT2G13660	unknown protein; [Source:TAIR;Acc:AT2G13660] Related with mitochondrion/nucleus
AT1G51430	unknown protein; BEST Arabidopsis thaliana protein match is (TAIR:AT3G28370.1); [Source:TAIR;Acc:AT1G51430] Related with cloroplast/cytoplasm
AT5G44980	F-box/RNI-like/FBD-like domains-containing protein [Source:TAIR;Acc:AT5G44980]
AT5G23830	MD-2-related lipid recognition domain-containing protein [Source:TAIR;Acc:AT5G23830] Related with Expression in the Root apex
TPS08 (AT4G20210)	Terpenoid cyclases/Protein prenyltransferases superfamily protein [Source:TAIR;Acc:AT4G20210] Related with Expression in the Root apex and Cloroplast
AT5G42460	F-box and associated interaction domains-containing protein [Source:TAIR;Acc:AT5G42460]
AT5G47260	ATP binding;GTP binding;nucleotide binding;nucleoside-triphosphatases [Source:TAIR;Acc:AT5G47260] Related with Defense response / mitochondrion
CPuORF24 (AT5G45428)	conserved peptide upstream open reading frame 24 [Source:TAIR;Acc:AT5G45428] TM protein domains
